# RANKL/OPG Axis and Bone Mineral Density in Pediatric Inflammatory Bowel Disease

**DOI:** 10.3390/jcm14155440

**Published:** 2025-08-01

**Authors:** Mariusz Olczyk, Agnieszka Frankowska, Marcin Tkaczyk, Anna Socha-Banasiak, Renata Stawerska, Anna Łupińska, Zuzanna Gaj, Ewa Głowacka, Elżbieta Czkwianianc

**Affiliations:** 1 Department of Paediatrics, Immunology and Nephrology, Polish Mother’s Memorial Hospital Research Institute, Medical University of Lodz, 90-419 Lodz, Poland; agnieszka.frankowska@stud.umed.lodz.pl (A.F.); marcin.tkaczyk@umed.lodz.pl (M.T.); 2 Department of Gastroenterology, Allergology and Pediatrics, Polish Mother’s Memorial Hospital Research Institute, 93-338 Lodz, Poland; sochabanasiak@gmail.com (A.S.-B.); elcia@friend.pl (E.C.); 3 Department of Endocrinology and Metabolic Diseases, Polish Mother’s Memorial Hospital-Research Institute, Department of Paediatric Endocrinology, Medical University of Lodz, 93-338 Lodz, Poland; renata.stawerska@umed.lodz.pl (R.S.); anna.lupinska@umed.lodz.pl (A.Ł.); 4 Center of Medical Laboratory Diagnostics and Screening, Polish Mother’s Memorial Hospital-Research Institute, 93-338 Lodz, Poland, ewa.biol@interia.pl (E.G.)

**Keywords:** pediatric inflammatory bowel disease, RANKL/OPG axis, bone metabolism, bone mineral density, ulcerative colitis, Crohn’s disease

## Abstract

**Background**: Inflammatory bowel diseases (IBD), such as Crohn’s disease (CD) and ulcerative colitis (UC), may impair bone metabolism, particularly in children. The RANKL/OPG axis, as a key regulator of bone turnover, may contribute to these disturbances. However, data in the pediatric population remain limited. **Methods**: A single-center, prospective observational study included 100 children aged 4–18 years, with a comparable number of girls and boys. Among them, 72 had IBD (27 CD, 45 UC) and 28 were healthy controls. Anthropometric, biochemical, and densitometric assessments were performed, including serum levels of RANKL and OPG, and markers of inflammation and bone turnover. **Results**: Children with CD had significantly lower height and weight percentiles compared to UC and controls. Serum RANKL and the RANKL/OPG ratio were significantly elevated in IBD patients, particularly in CD (*p* < 0.01). Total body BMD Z-scores were lower in IBD compared to controls (*p* = 0.03). Low BMD was found in 14.7% of UC and 26.3% of CD patients. In both groups, over 30% had values in the “gray zone” (−1.0 to −2.0). A positive correlation was observed between height and weight and bone density (*p* < 0.01). Higher OPG was associated with lower body weight (*p* < 0.001), while increased RANKL correlated with osteocalcin (*p* = 0.03). Patients receiving biological therapy had significantly lower BMD. **Conclusions**: Pediatric IBD is associated with significant alterations in the RANKL/OPG axis and reduced bone density. These findings support early screening and suggest RANKL/OPG as a potential biomarker of skeletal health.

## 1. Introduction

Inflammatory bowel diseases (IBD), such as Crohn’s disease (CD) and ulcerative colitis (UC), are persistent, relapsing inflammatory disorders of the gastrointestinal (GI) tract. The incidence of these diseases is increasing among pediatric patients [1]. IBD in children leads to intestinal inflammation and interferes with normal growth patterns, often causing delayed puberty and skeletal development disorders [2]. When it occurs during key periods of bone mass accumulation, it may result in osteopenia, osteoporosis, or an elevated risk of fractures later in life. Low bone mineral density (BMD) is frequently observed among children with IBD [3]. Although the precise mechanisms remain unclear, several molecular pathways regulating bone turnover are believed to be involved. In particular, the osteoprotegerin (OPG) and receptor activator of nuclear factor kappa-B ligand (RANKL) system has emerged as a significant area of interest.

In pediatric IBD, bone metabolic disorders arise from multiple factors, including nutritional deficiencies, hormonal dysregulation, and chronic systemic inflammation. A key role in this process is played by pro-inflammatory cytokines released during active disease. Cytokines such as interleukin-6 (IL-6), interleukin-1β (IL-1β), tumor necrosis factor-alpha (TNF-α), and interleukin-17A (IL-17A) contribute significantly to mucosal inflammation and tissue damage [4]. These cytokines also disrupt bone and muscle metabolism by stimulating RANKL expression and suppressing OPG production, thereby promoting osteoclast activity and bone resorption. Moreover, IL-17A inhibits osteoblast differentiation, further promoting bone loss [5].

**Figure 1:**
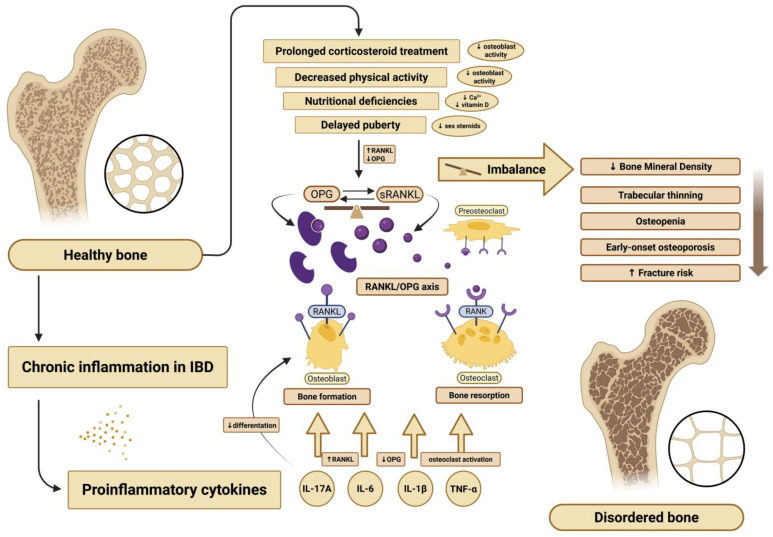
Pathophysiological mechanisms underlying bone impairment in pediatric IBD (created with BioRender.com).

OPG and RANKL, members of the tumor necrosis factor (TNF) superfamily, are central regulators of osteoclastogenesis [6]. RANKL promotes osteoclast formation, while OPG acts as a decoy receptor, blocking RANKL from binding to its receptor on osteoclast precursors. The RANKL/OPG balance reflects the state of bone remodeling, and its proper regulation is essential for maintaining bone density and structural integrity. While this pathway has been extensively studied in adults with IBD [7], data in pediatric populations remain limited and inconclusive. A graphical overview of these mechanisms is presented in Figure 1.

Children with IBD are exposed to multiple risk factors for skeletal complications, including malnutrition, prolonged corticosteroid therapy, and chronic inflammation [3,8,9]. Inflammatory cytokines released during disease flares—particularly IL-6, TNF-α, IL-1β, and IL-17A—contribute to systemic responses extending beyond the intestinal tract, affecting bone and muscle health [10,11]. These may manifest as bone metabolic disorders such as osteopenia, which can progress to early-onset osteoporosis [12,13].

The aim of this study is to deepen our understanding of the mechanisms contributing to bone abnormalities associated with dysregulation of the RANKL/OPG axis in children with IBD. Research into these mechanisms may support the development of targeted interventions to reduce long-term skeletal complications.

## 2. Materials and Methods

### 2.1. Study Design and Setting

**Figure 2:**
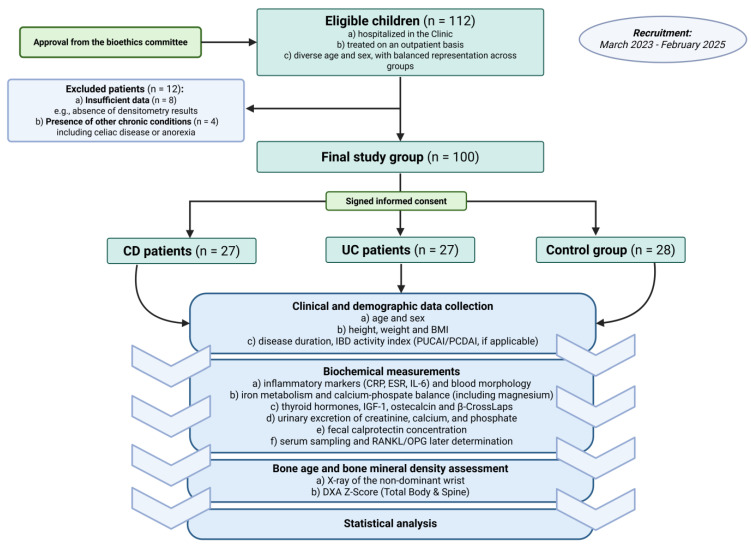
The flowchart of the study (created with BioRender.com).

We conducted a single-center, prospective, observational study involving pediatric patients hospitalized from 31 March 2023 to 26 February 2025 at the Department of Gastroenterology, Allergology, and Pediatrics of the Polish Mother’s Memorial Hospital Research Institute in Lodz. The study was approved by the Bioethics Committee (RNN/09/23/KE, 10 January 2023). Figure 2 presents a detailed flowchart illustrating patient selection and data collection procedures.

### 2.2. Participants

Inclusions criteria were a diagnosis of CD or UC based on clinical presentations, endoscopic findings, and histopatological examination of intestinal mucosal biopsies. The control group consisted of participants with no history of chronic illness and no use of medications that might affect laboratory findings.

Out of the 112 patients, 12 were excluded due to insufficient data (*n* = 8), including the absence of densitometry results (caused by failure to attend the scheduled examinations by patients), as well as the presence of chronic conditions (*n* = 4) identified during the study period (such as celiac disease or anorexia) that could influence the reliability of the findings. Patients with congenital bone disorders, such as osteogenesis imperfecta, were excluded from the study at the recruitment stage.

The control group was selected to ensure a comparable distribution of age and sex relative to the IBD group. Although no formal matching was applied, age and sex distributions did not differ significantly between the groups. The sample size was not calculated in advance but was considered adequate for exploratory analysis based on the available population during the study period.

Written informed consent was obtained from all parents or legal guardians. They were fully informed that participation was voluntary and that they could withdraw their child from the study at any time without any consequences.

All patients included in the study received treatment consistent with current clinical guidelines for IBD management. Each patient’s treatment history was reviewed, and participants were categorized into subgroups based on whether they had previously received or were currently receiving specific therapies—such as biological treatment, corticosteroids, or immunosuppressive drugs—or had not yet undergone any pharmacological treatment.

### 2.3. Clinical and Demographic Data

The final study group consisted of 100 children aged 4–18 years (mean age 13.70, SD ± 3.50, median 14), including 48 girls (mean age 12.92, SD ± 3.65, median 13) and 52 boys (mean age 14.44, SD ± 3.20, median 15). Patients diagnosed with UC accounted for 45% (45/100), CD—27% (27/100), and the remaining 28% (28/100) constituted the control group. Among patients with UC, 44.4% (*n* = 20) had clinical remission, 26.7% (*n* = 12) had mild disease, 15.6% (*n* = 8) had moderate disease, and 11.1% (*n* = 5) had severe disease based on the PUCAI scores. For those with CD, 55.6% (*n* = 15) had clinical remission, 25.9% (*n* = 7) had mild disease, 3.7% (*n* = 1) had moderate disease, and 14.8% (*n* = 4) had severe disease, according to the PCDAI scores. Among the patients with IBD, the average disease duration was approximately one year (1.63 years in CD and 0.82 years in UC, respectively). The majority of patients received systemic or oral corticosteroid therapy (75%, *n* = 54) and were treated with mesalazine (66.7%, *n* = 48). Thiopurines were used in 36% of patients (*n* = 26). Among biological agents, infliximab was the most commonly administered (23.6%, *n* = 17), followed by adalimumab and vedolizumab (both 12.5%, *n* = 6), and ustekinumab (1.4%, *n* = 1). The data are presented in Table 1.

In the control group, 46% (*n* = 13) were boys and 54% (*n* = 14) were girls. The mean age at examination was 13.31 years (SD ± 3.88) and did not differ significantly from that of the IBD group. Anthropometric data, including body weight, height, and BMI, were collected and subsequently plotted on appropriate percentile charts.

### 2.4. Biochemical Measurements

Inflammatory markers such as C-reactive protein (CRP) and erythrocyte sedimentation rate (ESR) were assessed. Laboratory tests included blood morphology parameters (hemoglobin concentration, red blood cell count, platelet count, and mean corpuscular volume), markers of iron metabolism (serum iron and ferritin), calcium-phosphate balance (total calcium and phosphate levels, parathyroid hormone [PTH], vitamin D, and alkaline phosphatase), as well as magnesium and creatinine concentrations, thyroid hormones, and fecal calprotectin concentration. Furthermore, the analysis included serum concentrations of interleukin-6 (IL-6), insulin-like growth factor 1 (IGF-1), osteocalcin, and β-CrossLaps. Urinary concentrations of creatinine, calcium, and phosphate were also measured. All tests were performed at the Polish Mother’s Memorial Hospital in Łódź using standardized methods. Osteocalcin and Crosslaps were measured by electrochemiluminescence immunoassay (ECLIA) on a Roche Cobas analyzer (Roche Diagnostics, Manheim, Germany). Additional blood samples were collected, and after an adequate number of samples had been obtained, concentrations of total RANKL and total OPG were determined using the ELISA method. During the sample collection period, all specimens were stored at approximately −20 °C, in accordance with the manufacturers’ recommendations regarding sample storage conditions. Vitamin D deficiency and insufficiency were defined as 25 (OH)D concentrations below 20 ng/mL (50 nmol/L) and between 21 and 29 ng/mL (52.5–72.7 nmol/L), respectively [14].

### 2.5. Bone Mineral Density and Bone Age Assessment

Bone mineral density (BMD) was assessed by dual-energy X-ray absorptiometry (DXA) using the Hologic Horizon system (Hologic Inc., Marlborough, MA, USA; MAN-04871-3402, version 007). For each patient, Z-scores were obtained for Total Body and Spine. A Z-score above −1.0 was considered normal, scores between −1.0 and −2.0 indicated mildly reduced BMD (the “gray zone”), while values below −2.0 were classified as low BMD. Only laboratory parameters obtained during a specific hospitalization or follow-up visit were considered; DXA scans were performed within one month of sample collection at the latest, except for patients whose scans were performed later or who missed the appointment—these cases were excluded from the analysis. In patients whose parents provided informed consent, an X-ray of the non-dominant wrist was performed to assess bone age.

### 2.6. Statistical Analysis

All statistical analyses were performed using the Statistica software, version 13.3 (TIBCO Software Inc. (2017), Mumbai, Maharashtra, India). The distribution of variables was assessed using the Shapiro–Wilk test. As most variables were not normally distributed, non-parametric tests were applied. Correlations were analyzed using Spearman’s rank test, while group comparisons were performed using the Mann–Whitney U test or the Kruskal–Wallis test with appropriate post hoc analysis. A *p*-value of <0.05 was considered statistically significant.

## 3. Results

### 3.1. Comparison of Clinical and Biochemical Parameters in Children with CD, UC, and Controls

Statistical analysis showed that children with CD had significantly lower percentile values for both height and weight, as well as a lower BMI, compared to other groups (*p* = 0.004, *p* = 0.01, and *p* = 0.04, respectively). The median height percentile in CD was 26.5 (IQR 35) and weight percentile 27 (IQR 37.5), while in UC patients these values 53 (IQR 56) and 55 (IQR 41), and in the control group 45 (IQR 51) and 45 (IQR 60.75), respectively.

C-reactive protein (CRP) levels were significantly higher in children with CD than in UC patients and in the controls (*p* = 0.046). Serum vitamin D3 concentrations were generally reduced in all groups. The median values were 26.8 ng/mL (IQR 6.3) in CD, 28.1 ng/mL (IQR 11.4) in UC, with the lowest levels observed in controls (23.1 ng/mL, IQR 15.27). However, this difference was not statistically significant (*p* = 0.28).

Platelet count was statistically higher in IBD patients compared to controls (*p* = 0.001). Although anemia appeared more frequently in IBD groups, this difference did not reach statistical significance (*p* = 0.05). UC patients demonstrated lower median values of alkaline phosphatase (ALP) and osteocalcin compared to other groups (*p* = 0.002, *p* = 0.001, respectively). The median vitamin D3 concentration in all groups was below the normal range, but no statistically significant differences were found between the groups (*p* = 0.28). Children with CD showed lower urinary calcium excretion (*p* = 0.044), and slightly lower serum calcium levels, although the latter was not statistically significant (*p* = 0.054). Median fecal calprotectin concentrations in stool were markedly different: 396 mg/kg in CD, 1100 mg/kg in UC, and 15 mg/kg in controls (*p* < 0.001). The data are presented in Table 2.

### 3.2. Gender Differences

Sex-related differences in the studied parameters were also identified within the study group. Among boys, statistically higher values were observed for ferritin (*p* = 0.002), hemoglobin (*p* = 0.00004), B-CrossLaps (*p* = 0.0002), and densitometric parameters in the Total Body projection (*p* = 0.049). Erythrocyte sedimentation rate was significantly lower in boys compared to girls (*p* = 0.001).

### 3.3. Differences Between Newly Diagnosed and Longstanding IBD

In our study, we identified some differences depending on disease duration. Newly diagnosed patients presented significantly higher levels of inflammatory markers (such as CRP, ESR, IL-6, and fecal calprotectin) and platelet count (all with *p* < 0.05). Anemia was statistically more frequent, and lower concentrations of osteocalcin were observed in this group (*p* = 0.01 and *p* = 0.001, respectively).

### 3.4. Bone Mineral Density

**Figure 3:**
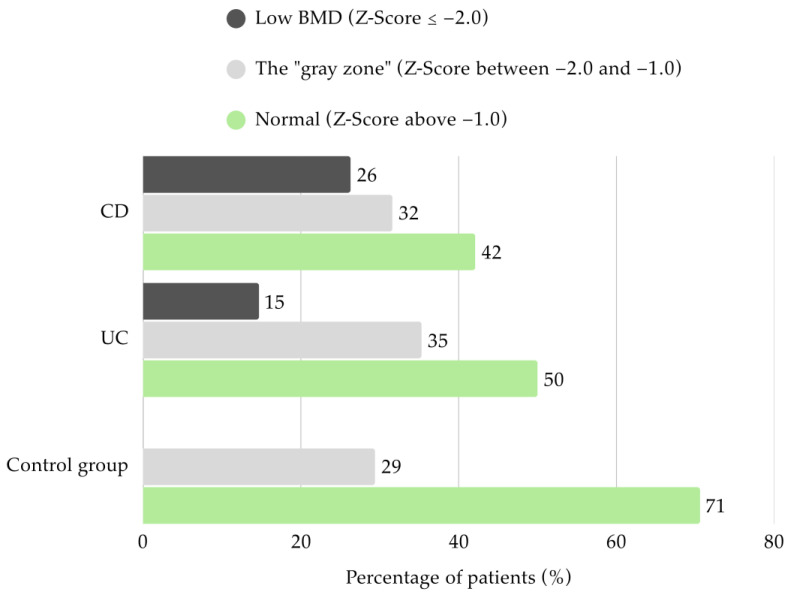
Bone mineral density Z-score in children with Crohn’s disease (CD), ulcerative colitis (UC), and controls.

Densitometry parameters were assessed in the majority of the study group (*n* = 70). Only half of the UC patients had BMD within the normal range in both DXA projection (*n* = 17). Among these children, 14.7% (*n* = 5) were classified with low BMD, while 35.3% (*n* = 12) was found to have mildly reduced BMD. In CD patients, 42.1% (*n* = 8) presented Z-Scores above −1.0. More than a quarter had low BMD (26.3%; *n* = 5) and 31.6% (*n* = 6) were categorized in the “gray zone”. None of the patients from the control group had low BMD, and 70.6% (*n* = 12) showed appropriate values (Figure 3).

**Figure 4:**
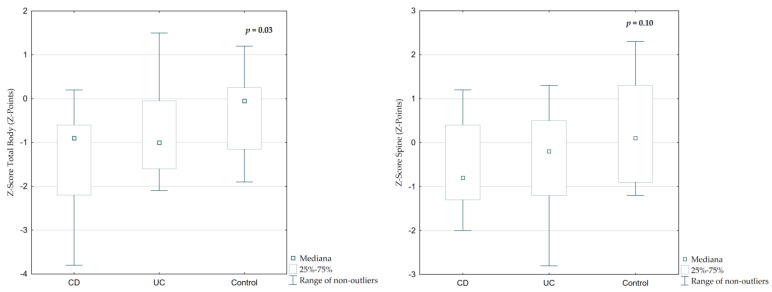
Differences in total body and spine Z-scores among children with CD, UC, and the control group.

The median Z-Score Total Body (TB) was −0.9 (IQR 1.3) in CD and −1.0 (IQR 1.53) in UC patients, while in controls it was significantly higher at −0.05 (IQR 1.35; Figure 4, *p* = 0.02). A similar trend was observed in Z-Score Spine (SP), which was lower in IBD patients (CD: −0.8, UC: −0.2) compared to controls (+0.1), though the difference was not statistically significant (Table 2, *p* = 0.10).

**Figure 5:**
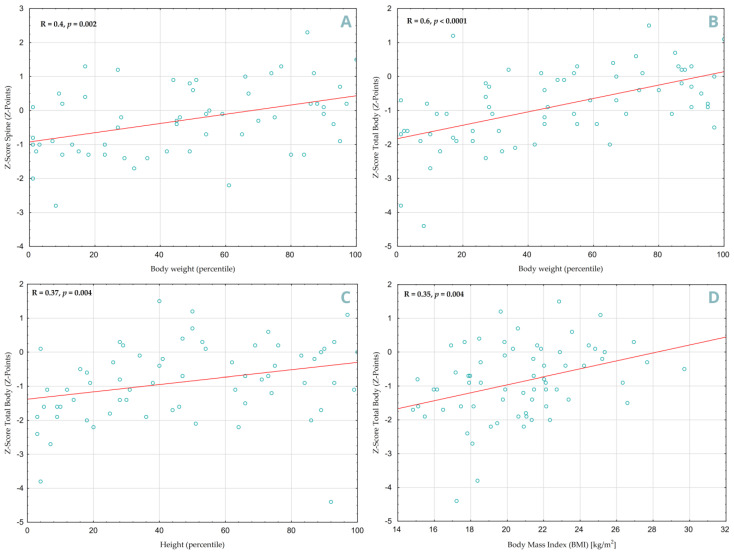
Correlation between anthropometric parameters and bone mineral density in children with IBD. (**A**)—Body weight percentile vs. Z-score Spine, (**B**)—Body weight percentile vs. Z-score Total Body, (**C**)—Height percentile vs. Z-score Total Body, (**D**)—BMI vs. Z-score Total Body. Individual data points from patients are marked in blue, with the red line indicating the overall distribution trend.

A positive correlation was found between increased body weight and higher Z-Score values in both SP (Figure 5A, R = 0.4, *p* = 0.002) and TB (Figure 5B, R = 0.60, *p* < 0.0001) projections. Height percentiles also showed a significant correlation, but only in the DXA TB projection (Figure 5C, R = 0.37, *p* = 0.004), similar to Body Mass Index (Figure 5D, R = 0.35, *p* = 0.004). Children with less delayed bone age had better densitometry results in both projections (TB: R = 0.51, *p* = 0.0002, SP: R = 0.36, *p* = 0.01). However, no statistically significant association was found between vitamin D3 concentration and TB Z-score (*p* = 0.18) or SP (*p* = 0.13).

In the adjusted multivariate analysis, lower Total Body Z-scores were significantly associated with older age and higher β-CrossLaps levels (*p* = 0.028 and *p* = 0.043). For lumbar spine BMD, boys had higher Z-scores than girls (*p* = 0.048). Other variables, such as BMI, IGF-1, and bone age, were not significantly related to bone mineral density at either site.

### 3.5. RANKL/OPG Axis

**Figure 6:**
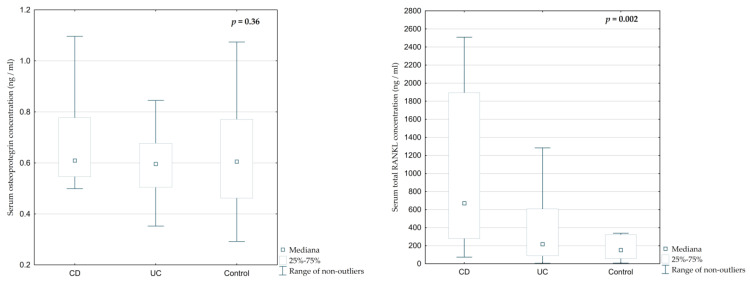
Differences in the serum concentration of total RANKL and osteoprotegerin among children with CD, UC, and the control group.

**Figure 7:**
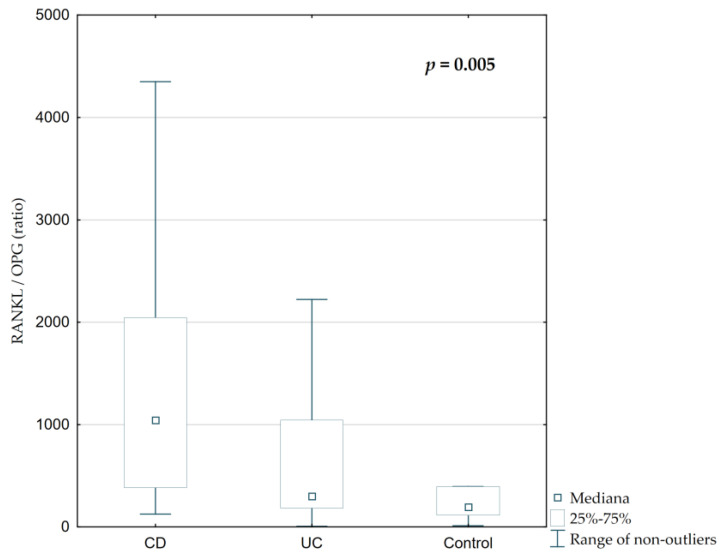
Differences in the RANKL/OPG ratio among children with CD, UC, and the control group.

Total serum RANKL concentration and RANKL/OPG ratio were significantly elevated in IBD patients, particularly those with CD (Figure 6 and Figure 7, *p* = 0.002 and *p* = 0.005, respectively). The median RANKL concentration in CD was 808.62 ng/mL, compared to 231.95 ng/mL in UC and 182.57 ng/mL in controls. The RANKL/OPG ratio was highest in CD (1059.27), followed by UC (336.89) and controls (243.38), with statistically significant differences between groups (Figure 7, *p* = 0.005). However, serum osteoprotegerin concentrations were similar across all groups (Table 2, Figure 6, *p* = 0.36).

Statistical analysis showed that higher serum concentration of osteoprotegerin was noted in patients with lower body weight (R = −0.4, *p* = 0.0004). A positive correlation was observed between vitamin D3 levels and OPG (R = 0.28, *p* = 0.01), RANKL (R = 0.32, *p* = 0.005) and the RANKL/OPG ratio (R = 0.26, *p* = 0.03). Higher serum osteocalcin concentrations and platelet count were associated with increased RANKL values (R = 0.25, *p* = 0.03; R = 0.26, *p* = 0.02, respectively), but were not significantly correlated with OPG. Moreover, a significant negative correlation was observed between IL-6 and osteocalcin concentrations (R = −0.26, *p* = 0.03). However, red blood parameters such as RBC, HGB and HCT were inversely correlated with OPG concentration (R = −0.25, *p* = 0.03; R = −0.31, *p* = 0.006; R = −0.31, *p* = 0.005, respectively). Correlation between RANKL/OPG ratio and DXA parameters, did not reach a statistical significance (TB: *p* = 0.69; SP: *p* = 0.47).

### 3.6. The Effect of Previous Treatment

In our study, steroid therapy affected DXA SP results, although the difference was not statistically significant (*p* = 0.07). Nevertheless, in the adjusted multivariate model, prior steroid use was significantly associated with lower lumbar spine Z-scores (β = −1.27, *p* = 0.036). Previous use of mesalazine was associated with higher vitamin D3 levels (*p* = 0.02), and lower alkaline phosphatase and osteocalcin concentrations (*p* = 0.01 and *p* = 0.03, respectively). Treatment with azathioprine correlated with lower fecal calprotectin (*p* = 0.049) and ESR (*p* = 0.049) values observed at follow-up.

Statistically, prior biological treatment with adalimumab and infliximab was linked to decreased BMD in the TB projection (*p* = 0.02 and *p* = 0.01, respectively). Infliximab use was further associated with elevated ESR (*p* = 0.001 and platelet count (*p* = 0.03).

## 4. Discussion

The influence of Crohn’s Disease and Ulcerative Colitis on bone metabolism among the pediatric population is undeniable [11]. Nevertheless, this aspect is frequently neglected during the course of diagnosis and therapeutic process of inflammatory bowel diseases. In light of this, the analysis aims to emphasize the significance of the regular bone assessments in pediatric IBD, in order to prevent future skeletal complications.

Prior to exploring the biochemical parameters of bone metabolism, basic anthropometric ones such as weight and height should also be assessed. According to our study and the work by Rudra et al. both decreased BMI and height were observed among patients with IBD [3]. Reduction in these parameters, as well as the chronic steroid therapy may be the first visible and easily measurable prognostic indicators for bone metabolic disorders. From a clinical perspective, routine assessment of height and weight in pediatric patients may represent a practical and cost-effective approach for the early identification of individuals at increased risk of developing bone disorders, particularly from the standpoint of pediatricians and gastroenterologists. In the other analysis, made by Hwa Jong Kim et al., it was also strongly highlighted that early diagnosis established before the age of 10, higher levels of CRP, and long-term steroid therapy indicated significance decreased in bone mineral density [15]. Therefore, early diagnosis and regular monitoring, combined with patient and family education, are crucial for effective disease management. Thus, our study highlights the importance of multidisciplinary healthcare among patients with IBD, especially those who are in early stages of disease or undergoing steroid therapy.

The next key parameter assessed was CRP, which has been reported to be more frequently elevated in children with Crohn’s disease than in those with ulcerative colitis [16]. In addition, the CRP is associated with vitamin D levels. It has been noticed that this micronutrient supplementation may cause a decline in the CRP levels, which can provide a highly accessible method to contribute to reducing the concentration of the inflammatory protein [17]. This approach helps achieve two goals simultaneously because among all the examined patients, the level of vitamin D was reduced. This underscores the importance of vitamin D supplementation, not only for maintaining bone health but also for modulating the potential inflammatory processes. Therefore, regular monitoring and correction of vitamin D deficiency may serve as a simple adjunctive strategy in comprehensive disease management.

Some differences have also been observed among particular sexes. According to our study, higher levels of hemoglobin, ferritin, B-CrossLaps, and bone density were noted in the boys. Contrary tendency has been documented in the ESR concentration. Such results may be caused by the elevated testosterone level, which supports improved bone density and stimulates erythropoiesis [18,19]. In contrast, decreased levels of ESR may be indicated by the testosterone’s anti-inflammatory action [20].

Analyzing basic morphological parameters may also yield some information about the stage of disease development. The study of Daniluk et al. aligns with the results obtained in the present study [21]. Among patients, recently diagnosed variables such as platelets, ESR, and calprotectin were highly elevated as a result of the chronic inflammation, whereas the hemoglobin and MCV were diminished due to blood loss. In addition, in the group of examined patients, the elevated IL-6 and serum phosphate levels were also noted. These variables are consistent with the inflammatory profile typically observed during active phases of IBD. These results emphasize the importance of assessing hematological and inflammatory parameters to determine disease activity and identify patients at greater risk for extraintestinal complications, including bone loss. Future prospective studies should further validate composite biomarker panels for bone risk stratification in IBD.

A diagnostic method that should significantly be considered among pediatric patients with IBD is densitometry. Children with CD or UC often have decreased height and body weight values relative to age- and sex-specific percentile standards [22]. Proper measurements are crucial for physiological bone development, which are associated with improved bone density. Studies show that children with IBD have lower values of BMD in comparison to age-matched controls [8]. Therefore, early and regular examination may help to prevent bone disorders or early diagnosis may arrest the progression or indicate its remission. Furthermore, our study shows that patients whose bone age was more closely to their chronological one, are most likely to present better densitometric outcomes. It may suggest that bone age delay, which is often a consequence of chronic inflammation and poor disease control, can notably interfere with BMD interpretation in pediatric IBD. Therefore, it is advisable to assess and interpret densitometry results in relation to skeletal maturation, rather than relying on chronological age, similarly to what has already been demonstrated in other studies [23]. These observations have direct clinical implications—the bone age determination should be considered in addition to DXA during the assessment of bone health among IBD patients. Accounting for skeletal maturity when interpreting bone mineral density Z-scores may enhance diagnostic accuracy and guide treatment.

The need for such an individualized approach to bone health evaluation is further supported by our multivariate analysis, which showed that older age and higher β-CrossLaps levels were independently associated with lower Total Body Z-scores. For the lumbar spine, male sex was linked to better outcomes, while steroid therapy was significantly associated with reduced bone mineral density. This finding aligns with the well-documented effect of glucocorticosteroids in suppressing osteogenesis and accelerating bone resorption. Similarly, Ward et al. reported rapid, spine-predominant bone loss and a high prevalence of asymptomatic vertebral fractures in children undergoing long-term corticosteroid therapy [24]. These findings underscore the need for comprehensive and individualized bone health assessment in children with IBD, particularly those receiving corticosteroids.

Another important aspect worth analyzing are the direct markers of bone metabolism such as RANKL and osteoprotegerin. A recent study suggests including the measurements of RANKL/OPG to the molecular investigations among patients with IBD [25]. Correlation between both parameters and bone metabolic disorders are widely described within the literature [26,27]. However, some associations that may have been previously overlooked demonstrated statistical significance in our study. An increase in OPG has been observed among the children with low body weight. This correlation may be due to elevated OPG levels functioning as a defense mechanism against excessive bone resorption. In light of these results, the potential of RANKL/OPG as biomarkers for the early identification of bone remodeling abnormalities should be emphasized.

The modulatory role of vitamin D in the RANKL/OPG axis is complex and occasionally contradictory. There are some studies in which the proposed hypotheses differ, and the results are not always consistent. One of these observed correlations was described by the Czech scientists, who noted a rise in OPG concentration concomitant with elevated vitamin D levels [28]. Research shows that this vitamin may lead to the rise in the OPG, especially in inflammatory conditions, as an element of the vitamin D anabolic effect. A similar finding was noted in our study. Conversely, there are some other studies, including ours that show that vitamin D can stimulate the expression of the RANKL in osteoblasts while simultaneously suppressing OPG synthesis in the osteoblasts, which is the natural inhibitor of RANKL [29]. Thus, it has a modulatory effect on the RANKL/OPG ratio. Additionally, vitamin D helps maintain bone density by supporting calcium and phosphate absorption. Consequently, in the presence of adequate levels of calcium and phosphate, vitamin D may modulate the RANKL/OPG axis in a physiological manner. Nonetheless, children with IBD consistently present with reduced vitamin D levels, which may directly affect their bone mineral density. According to numerous studies, it has been demonstrated that early vitamin D supplementation significantly improves densitometric results among this part of the population [30,31].

In addition, the relation that is also worth pointing out is the correlation between the RANKL/OPG ratio and concentrations of osteocalcin and platelets. The results obtained in our investigation demonstrate that among patients with higher RANKL/OPG ratio, elevated osteocalcin activity and platelet count was observed. Increased RANKL/OPG ratio suggests enhanced bone remodeling, which also includes osteoblasts activation process thus it leads to elevation in osteocalcin production. Furthermore, studies confirm that megakaryocytes can produce RANKL and influence their proliferation [32,33]. RANKL has a stimulatory effect on bone marrow, in parallel, a lower concentration of OPG indicates its unrestricted activity leading to increased platelet production. The exact same effect is achieved by chronic inflammation, which results in reactive thrombocytosis. Due to these mechanisms, among our patients both parameters were elevated as a manifestation of the active bone marrow and inflammation. From the clinical perspective, regular assessment of platelet count may provide indirect insight into evaluation of bone turnover and inflammatory bone resorption. This area remains insufficiently explored and warrants further investigation.

Most of the patients included in our study were undergoing anti-inflammatory or biological therapy. Therefore, some correlations between the medication and particular parameters need to be pointed out. First of all, it has been noticed that there is an association between steroids and increased calprotectin concentration. They were used mostly during the high activity of the disease. Accordingly, elevated calprotectin levels may reflect a recent exacerbation of the disease. Moreover, the GKS inhibits osteogenesis, which explains the reduced spine density observed in the studied population [3]. Another relevant association that deserves attention is the one observed between mesalazine in the past and reduced levels of bone markers such as alkaline phosphatase and osteocalcin. This may indicate a suppressive effect of mesalazine on osteoblast activity. In patients undergoing treatment with the use of infliximab, elevated platelet counts and ESR values, along with decreased DXA TB, have been observed. Similar associations with reduced bone density were also noted in patients treated with adalimumab. This may be caused by high inflammatory activity, hence higher levels of inflammatory markers and reduced bone density, which are more commonly seen in individuals qualifying for biological therapy. It may suggest that patients receiving this type of treatment are most likely to suffer from a more severe course of the disease and require intensified treatment, which should be taken into account in future clinical decisions and highlights the importance of regular monitoring in this group. However, due to a small sample size, caution is required when interpreting its results.

These findings may support clinical changes that simplify and accelerate the diagnostic pathway in children with IBD by incorporating basic parameters reflecting bone metabolism and inflammatory activity. Future investigations should examine the longitudinal effects of specific medications on bone turnover to guide the safest therapeutic strategies and optimize bone health outcomes.

This study has several strengths. It provides prospective data from a well-defined pediatric cohort, collected in a clinical setting during routine hospital care. The analysis includes both laboratory markers (serum RANKL and OPG) and bone mineral density assessment, reflecting real-world diagnostic and monitoring practices. This combination enhances the clinical relevance of the findings and supports their potential application in everyday pediatric IBD management. However, some limitations should be acknowledged. First, the study was conducted in a single center, which may limit the external validity. Second, the relatively small sample size may reduce the statistical power for subgroup analyses. Finally, the observational nature of the study precludes causal inference, and some potential confounding factors (such as physical activity or vitamin D supplementation) could not be fully controlled.

## 5. Conclusions

Our study confirms that metabolic bone disorders are a frequent complication in pediatric IBD, especially in Crohn’s disease. Reduced bone mineral density is associated with RANKL/OPG axis disturbances—including elevated serum RANKL levels and an increased RANKL/OPG ratio—both correlating with disease activity and bone turnover markers. Low BMI, delayed bone age, and prolonged corticosteroid therapy are strong predictors of impaired bone status. These findings highlight the importance of minimizing long-term steroid use where possible and considering early introduction of biologic therapy in appropriate cases. Consequently, routine DXA screening should be an integral part of comprehensive care in children with IBD, especially in severe cases. The RANKL/OPG axis may serve as both a diagnostic biomarker and a potential therapeutic target in the prevention of long-term skeletal complications in children with IBD.

## Figures and Tables

**Table 1:** Demographic and clinical characteristics of the study group.

	Crohn’s Disease (*n* = 27)	Ulcerative Colitis (*n* = 45)	Total IBD (*n* = 72)
**Gender, male *n*** (%)	14 (51.9)	25 (55.6)	39 (54.2)
**Age at diagnosis, yr, mean (SD)**	11.59 ± 3.19	13.32 ± 3.60	12.67 ± 3.53
**Age at examination, yr, mean (SD)**	13.13 ± 3.22	14.74 ± 3.17	14.14 ± 3.26
**Disease duration, yr, median (IQR)**	1.63 (2.54)	0.82 (1.94)	1.01 (2.17)
**Therapy (past or current)**			
Mesalazine *n* (%)	8 (29.6)	40 (88.9)	48 (66.7)
Corticosteroids *n* (%)	16 (59.3)	37 (82.2)	54 (75.0)
Thiopurine *n* (%)	13 (48.1)	13 (28.9)	26 (36.1)
Nutritional therapy *n* (%)	14 (51.9)	0 (0.0)	14 (19.4)
Infliximab *n* (%)	7 (25.9)	10 (22.2)	17 (23.6)
Adalimumab *n* (%)	3 (11.1)	3 (2.2)	6 (12.5)
Vedolizumab *n* (%)	1 (3.7)	5 (6.7)	6 (12.5)
Ustekinumab *n* (%)	0 (0.0)	1 (2.2)	1 (1.4)
**Disease Activity** **PCDAI ^1^/PUCAI ^2^**			
Remission *n* (%)	15 (55.6)	20 (44.4)	35 (48.6)
Mild *n* (%)	7 (25.9)	13 (26.7)	19 (26.4)
Moderate *n* (%)	1 (3.7)	7 (15.6)	8 (11.1e)
Severe *n* (%)	4 (14.8)	5 (11.1)	9 (12.5)

^1^ PCDAI—Pediatric Crohn’s Disease Activity Index, ^2^ PUCAI—Pediatric Ulcerative Colitis Activity Index.

**Table 2:** Comparison of clinical and biochemical parameters in Children with CD, UC, and controls.

Parameter	Classification of Study Group, Median (IQR)			*p*-Value
	Crohn’s Disease (*n* = 27)	Ulcerative Colitis (*n* = 45)	Control Group (*n* = 28)	
**BMI [kg/m^2^]**	19.28 ± 3.12 *	21.28 ± 3.39 *	19.78 ± 3.61 *	**0.04**
**Height** [percentiles]	26.50 (35.00)	53 (56.00)	45 (51.00)	**0.004**
**Weight** [percentiles]	27 (37.50)	55 (41.00)	45 (60.75)	**0.01**
**CRP ^1^** [mg/L]	↑ 1.16 ± 4.41 *	0.40 ± 1.37 *	0.11 ± 0.41 *	**0.046**
**ESR ^2^** [mm/h]	12 (17.5)	9 (24.0)	4 (8.75)	0.09
**Calcium** [mmol/L]	2.45 (0.11)	2.49 (0.10)	2.51 (0.16)	0.054
**Phosphate** [mmol/L]	1.52 (0.16)	1.39 (0.28)	1.42 (0.33)	0.56
**Magnesium** [mmol/L]	0.84 (0.07)	0.84 (0.06)	0.85 (0.09)	0.98
**Vitamin D** [ng/mL]	↓ 26.80 (6.30)	↓ 28.10 (11.40)	↓ 23.00 (15.27)	0.28
**Parathormone** [pg/mL]	25.55 (14.05)	22.00 (16.65)	23.50 (9.75)	0.38
**TSH** [ulU/mL]	1.94 (1.06)	1.71 (1.15)	2.10 (0.95)	0.30
**FT4** [ng/mL]	1.23 (0.30)	1.28 (0.26)	1.31 (0.23)	0.53
**RBC ^3^** [10^6^/μL]	4.71 (0.76)	4.56 (0.71)	4.75 (0.53)	0.43
**HGB ^4^** [g/dL]	12.25 (1.57)	12.80 (2.90)	13.45 (2.03)	0.05
**HCT ^5^** [%]	37.55 (4.83)	38.60 (7.00)	38.95 (5.35)	0.28
**MCV ^6^** [fL]	81.25 (4.18)	83.50 (10.40)	81.90 (6.75)	0.14
**PLT ^7^** [10^3^/μL]	317.50 (83.25)	333 (99.00)	258 (87.5)	**0.001**
**Ferritin** [ng/mL]	28.40 (24.12)	26.85 (31.75)	33.80 (15.50)	0.23
**Ferrum** [ug/dL]	45 (46.75)	41 (43.50)	41.50 (28.50)	0.90
**ALP ^8^** [U/L]	144 (126.75)	81 (58.00)	168 (120.00)	**0.002**
**IGF-1** [ng/mL]	245.20 (213.60)	279.10 (175.00)	329.50 (221.60)	0.62
**IL-6** [pg/mL]	5.20 (9.05)	6.90 (20.50)	3.30 (6.80)	0.29
**Osteocalcin** [ng/mL]	77.85 (74.35)	38.50 (31.60)	63.30 (50.75)	**0.001**
**Beta-CrossLaps** [ng/mL]	1375 (897.83)	1088 (682.60)	1227 (672.12)	0.25
**Creatinine (urine)** [mg/dL]	122.99 (195.74)	118.50 (120.44)	129.90 (131.11)	0.69
**Calcium (urine)** [mmol/L]	1.2 (2.73)	2.57 (2.61)	2.87 (1.64)	**0.044**
**Phosphate (urine)** [mmol/L]	23.05 (38.66)	21.39 (25.10)	29.06 (16.50)	0.23
**Fecal Calprotectin** [mg/kg]	↑ 396 (948)	↑ 1100 (1667.50)	15 (26)	**<0.001**
**Bone Age Deviation** [years]	−0.05 (2.1	+0.22 (1.87)	−0.10 (2.05)	0.677
**Z-Score TB ^9^** [Z-Points]	−0.90 (1.30)	−1.00 (1.53)	−0.05 (1.35)	**0.030**
**Z-Score Spine** [Z-Points]	−0.80 (1.50)	−0.20 (1.45)	+0.10 (1.95)	0.102
**RANKL** [ng/mL] **	808.62 (1770.56)	231.95 (536.88)	182.57 (1621.95)	**0.002**
**OPG** [ng/mL]	0.61 (0.19)	0.60 (0.17)	0.60 (0.30)	0.366
**RANKL/OPG** [ratio] **	1059.27 (2112.29)	336.89 (982.95)	243.38 (1600.25)	**0.005**

^1^ CRP—C-reactive protein, ^2^ ESR—erythrocyte sedimentation rate, ^3^ RBC—red blood cells, ^4^ HGB—hemoglobin, ^5^ HCT—hematocrit, ^6^ MCV—mean cell volume, ^7^ PLT—platelet count, ^8^ ALP—alkaline phosphatase, ^9^ TB—Total Body, *—the mean and standard deviation (SD) were used to better illustrate the distribution and variability, **—the eight patients with the highest values were excluded from the analysis to minimize the impact of extreme outliers, ↑—above normal range, ↓—below normal range. Reference ranges: CRP < 1 mg/L, ESR < 15 mm/h, calcium 2.25–2.75 mmol/L, inorganic phosphate 0.9–1.6 mmol/L, magnesium 0.8–1.0 mmol/L, vitamin D 30–50 ng/mL, parathormone 4.8–40 pg/mL, PLT 150–450 thousands/μL, IL-6 < 10 pg/mL, Z-Score TB and Z-Score Spine > −1.0 Z-Points, fecal calprotectin < 50 mg/kg. Reference ranges for the remaining parameters (TSH, FT4, RBC, HGB, HCT, MCV, ferrum, ferritin, ALP, IGF-1, osteocalcin, beta-CrossLaps) are age- and sex-dependent. Statistically significant differences are highlighted in bold.

## Data Availability

The datasets generated and analyzed during the current study are not publicly available due to personal data protection but are available from the corresponding author on reasonable request.
